# Keeping Athletes Healthy at the 2020 Tokyo Summer Games: Considerations and Illness Prevention Strategies

**DOI:** 10.3389/fphys.2019.00426

**Published:** 2019-04-17

**Authors:** Lauren C. Keaney, Andrew E. Kilding, Fabrice Merien, Deborah K. Dulson

**Affiliations:** ^1^Sports Performance Research Institute New Zealand, Auckland University of Technology, Auckland, New Zealand; ^2^AUT Roche Diagnostics Laboratory, Auckland University of Technology, Auckland, New Zealand

**Keywords:** Olympic, Paralympic, illness, strategies, health, stressors

## Abstract

Keeping athletes healthy will be important for optimal athletic performance at the 2020 Tokyo Summer Olympic and Paralympic Games. Athletes will be exposed to several stressors during the preparatory and competition phases of the Summer Games that have the potential to depress immunity and increase illness risk. This mini-review provides an overview on effective and practical stressor-specific illness prevention strategies that can be implemented to maintain and protect the health of Olympic and Paralympic athletes.

## Introduction

Acute illness is one of the single biggest factors that can prevent athletes from successful performance at pinnacle events (Raysmith and Drew, 2016). Upper respiratory tract symptoms (URTS) are the most common illness reported by elite athletes at the Olympic and Paralympic Games (Derman et al., 2013, 2017; Engebretsen et al., 2013; Soligard et al., 2017). Common URTS include sore throat, headache, runny nose and coughing (Walsh et al., 2011b). URTS can negatively impact training availability, reduce exercise performance, and can even result in athletes missing a major competition (Gleeson and Pyne, 2016). The importance of maintaining athlete health has been highlighted by studies demonstrating that World and Olympic winning medal athletes experience fewer URTS than less successful athletes (Raysmith and Drew, 2016; Svendsen et al., 2016). As such, keeping athletes healthy in preparatory and competition phases of the 2020 Tokyo Olympic and Paralympic Games (referred to as “Summer Games” in this mini review) will contribute toward optimal performance. The purpose of this mini review is to describe how stressors impact upon an athlete’s risk for illness and to identify which athletes may be more susceptible to illness at the Summer Games, along with outlining strategies to maintain and protect athlete health.

## Preparatory Phase of the Summer Games

Keeping athletes healthy in the lead up to the Summer Games is important for optimal performance. In elite track and field athletes, having fewer illnesses (and injuries) and completing more than 80% of planned trainings in the 6-months prior to a major event increases the likelihood of achieving pre-defined performance goals (Raysmith and Drew, 2016). Nevertheless, maintaining athlete health during the preparatory phase may prove to be a challenging task, as both Northern and Southern hemisphere athletes will be exposed to different stressors that can challenge immunity and increase URTS risk, including seasonal specific stressors, limited ultraviolet B exposure (UVB), heat acclimation and travel.

### Seasonal Specific Stressors

#### Seasonal Influenza

It is well established that the incidence of influenza exhibit seasonal fluctuations, with peak incidence occurring during the winter months (Doyle and Cohen, 2009). Therefore, compared to Northern hemisphere athletes, athletes residing in the Southern hemisphere will be at an increased risk for infectious URTS episodes during the preparatory phase of the Summer Games.

**Table 1:** Summary of five key illness prevention strategies that athletes should consider adhering to during the Summer Games.

Strategy	Proposed rationale	Practical recommendations	References
**1. Hygiene practices**	Minimize risk for infection transmission	- Hand hygiene: Wash hands regularly (rub hands with soap >20 s and dry hands thoroughly with clean towel) and carry alcohol-based hand gel	Schwellnus et al., 2016
		- Clean sporting equipment and clothing regularly	Keaney et al., 2018;Walsh, 2018
		- Isolate sick athletes and support staff (e.g., move out roommates)	
		- Avoid self-inoculation by not touching eyes, nose, and mouth	
		- Avoid shaking hands with other athletes and support personal	
		- Where possible, avoid crowded areas, sick people, young children (if avoidance is not possible, wear facial masks)	
**2. Maintain day-to-day CHO availability**	Preventing low CHO availability may minimize the exercise induced rise in stress hormones (cortisol and catecholamines) which in turn may attenuate immune perturbations	- Total CHO intake should match daily training and competition requirements	Burke et al., 2011;Bermon et al., 2017
		- Athletes engaging in prolonged continuous exercise or high intensity intermittent team sport exercise should aim to consume 30–60 gCHO/h	
			
**3. Probiotic supplementation**	Probiotics may help to reduce the incidence, severity, and duration of URTS	- Type: Non-refrigerated (travel friendly), multi-strain probiotic combining *Lactobacillus* and *Bifidobacterium*, ensure selected probiotic complies with WADA anti-doping regulations	Pyne et al., 2015;Williams et al., 2018
		- Dosage: 1 × 10^9^ colony forming units per day	
		- Timing: Commence probiotic supplementation at least 2 weeks before traveling to Tokyo, to allow adequate time for colonization	
		- Potential side effects: In first 2 weeks athletes may experience gastrointestinal issues (e.g., stomach rumbles, increased flatulence), athletes experiencing these symptoms should take their probiotic on an empty stomach. If side effects persist (>2 weeks) try reducing the dosage by half and gradually increase dosage as symptoms ease	
**4. Minimize stress and anxiety**	Stress and anxiety are risk factors for illness. Management of stress and anxiety may lower risk for URTS	- Identify athletes with high anxiety and stress using validated questions, e.g., Depression, Anxiety, Stress Scale (DASS-21), Recovery Stress Questionnaire (REST-Q-Sport-52)	Schwellnus et al., 2016;Drew et al., 2017;Walsh, 2018
		- Monitor stress and anxiety using a wellness questionnaire (refer to section “Strategies to Maintain Athlete Health During HA” for details)	
		- Consult a psychologist to provide education around anxiety and stress management techniques	
		- Mindfulness practice (refer to section “Strategies to Maintain Multi-Competition/Event Athlete Health” for details)	
**5. Improve sleep**	Sleep deprivation is a risk factor for illness. Improving sleep may reduce URTS risk	- Use objective (e.g., wrist actigraphy) or subjective (e.g., questionnaire) methods to identify sleep deprived athletes (<7 h per night) athletes	Fullagar et al., 2015;O’Donnell and Driller, 2017
		- Aim for a minimum 8 h of quality sleep per night	
		- Apply sleep hygiene strategies to optimize sleep quantity and quality [e.g., maintaining a regular bed and wake time, ensuring a quiet, cool, and dark bedroom environment (19–22°C), avoidance of stimulants (e.g., caffeine) prior to sleep, avoidance of light-emitting technology devices in the 30 min prior to sleep]	

##### Strategies to minimize seasonal influenza risk

• Vaccination: Advise athletes to have the influenza vaccine in autumn (April) before the influenza season as it usually takes 5–7 weeks to take effect (Schwellnus et al., 2016). Administration of the vaccine should occur during a non-competition period, or at least 2 weeks prior to competition to allow time for the development of a specific adaptive immune response and any potential side effects (Daly and Gustafson, 2011). There may be some benefit in performing moderate intensity exercise prior to vaccine administration as it has been shown to facilitate vaccine efficacy (Edwards et al., 2007, 2012) and reduce adverse reactions (Lee et al., 2018). This adjuvant strategy seems to be most successful in immunocompromised individuals (e.g., elderly) (Ranadive et al., 2014), therefore it is reasonable to suggest it may be worthwhile in elite athletes. It is considered a harmless strategy where the potential benefits could be crucial to preparation, however, further research on athletes is required to determine optimal protocols.
• Hygiene: Maintain good hygiene (refer to Table 1 for guidelines).
• Illness monitoring: Monitor illness to enable early detection and application of appropriate illness prevention strategies. Use the Jackson Common Cold Scale (Jackson et al., 1958) to monitor athlete illness. Consider monitoring household illness by adding a question alongside the Jackson Common Cold Scale (Keaney et al., 2018). Household illness monitoring is a promising strategy, although, further research in this area is required.
• Probiotic supplementation: Supplement throughout the preparatory phase (3 months) (refer to Table 1 for further guidelines).
• Zinc acetate supplementation: Supplement athletes experiencing acute URTS with zinc acetate lozenges (75 mg/day) to decrease the duration of URTS (Note: Zinc must be taken <24 h after onset of URTS and can be taken for 1–2 weeks) (Maughan et al., 2018). Excessive zinc supplementation (>150 mg/day) should be avoided as it can impair immune cell functions.

#### Cold Environmental Conditions

Upper respiratory tract symptoms can result from infectious (viral, bacterial, or fungal etiology) or non-infectious and inflammatory (e.g., caused by allergies, asthma and trauma to respiratory epithelial membranes) causes (Gleeson and Pyne, 2016). Southern hemisphere athletes training during winter will be exposed to cold dry air. Inhalation of cold dry air can damage airway epithelium and lead to non-infectious URTS episodes (Koskela, 2007). Athletes with asthma and allergies may be at higher risk for URTS, as winter training has been shown to increase URTS incidence among individuals with these conditions (Hyrkäs et al., 2014; Gleeson and Pyne, 2016).

##### Strategies to minimize cold air mediated non-infectious URTS

• Diagnose asthma and allergies: Administer the validated questionnaire Allergy Questionnaire for Athletes (AQUA) to identify athletes with asthma and allergies (Bonini et al., 2009). Confirm the diagnosis with a physician.
• Control asthma and allergies: Ensure appropriate therapeutic control of asthma and allergies and comply to World Anti-Doping Agency (WADA) regulations (Helenius and Haahtela, 2000).
• Protect airways: When practical, take extra precautions to avoid inhalation of cold dry air (below 0°C). For example, train indoors or for outdoor training use facial masks to protect airways (Walsh, 2018). It is unknown if facial mask reduce URTS incidence, however, they can attenuate cold air exercise-induced asthma which is known to elicit non-infectious URTS episodes (Beuther and Martin, 2006).

#### Summer Allergies and Asthma

Northern hemisphere athletes will be exposed to environmental factors and high periods of allergen load during the preparatory phase of the Summer Games, including heat and humidity, pollen, grasses, weed, mold, and dust. During exercise, high ventilation rates combined with increased exposure to environmental factors and allergens can exacerbate asthma and allergies. Prevalence of asthma and allergy is high in elite athletes, especially endurance athletes (Silva and Moreira, 2017). Exacerbations of asthma and allergies may elicit non-infectious URTS, such as runny nose, repetitive sneezing, and coughing, all of which can disrupt training and performance (Gleeson and Pyne, 2016).

##### Strategies to minimize summer allergy and asthma mediated non-infectious URTS

• Diagnose and control asthma and allergies (see section “Strategies to Minimize Cold Air Mediated Non-infectious URTS” for details).
• Allergen avoidance: When practical, avoid exposure to allergens (e.g., clean room and change bed lining regularly to reduce house dust mite exposure. Follow pollen forecasts and consider adapting training venues and training schedules during high pollen periods, etc.) (Silva and Moreira, 2017).

### Low Ultraviolet B Exposure

Southern hemisphere athletes will be at an increased risk for Vitamin D (VD) deficiency as it is more prevalent during winter when UVB exposure and endogenous synthesis of VD are low (Backx et al., 2017). Previous research suggests that up to 50% of athletes could be considered to have an inadequate VD status, during winter training months (He et al., 2013, 2016). Some Northern hemisphere athletes may also be at risk for VD deficiency, such as indoor athletes, athletes with dark skin tone, athletes who live and train in northern latitudes (<30° or >70°) and athletes residing in countries with a poor summer season with limited sun exposure (i.e., sun exposure <20 min/day) (He et al., 2016). VD deficiency appears to be an important determinant of URTS risk in athletes (He et al., 2013, 2016). Evidence suggests an optimal serum 25(OH)D of 75 nmol/L may enhance immunity and prevent URTS (He et al., 2016).

#### Strategies to Maintain VD Levels

• Vitamin D recommendations for Southern hemisphere and at-risk Northern hemisphere athletes: There may be some benefit in measuring athletes serum 25(OH)D concentration, to allow more targeted VD supplementation. As outlined in recent guidelines, athletes with serum 25(OH)D concentrations <75 nmol L^−1^ should be supplemented with 2000–4000 IU VD3/day (Owens et al., 2018). However, the measurement of serum 25(OH)D is not always feasible (cost approximately US$255 per athletes) and may not be the most appropriate measure of an athletes VD status (Allison et al., 2018; Owens et al., 2018). Therefore, rather than measuring serum 25(OH)D, the most practical approach may be to supplement all Southern hemisphere and at-risk Northern hemisphere athletes with 1000 IU VD3/day (comply to WADA anti-doping regulations) (He et al., 2016). There is some risk for toxicity when supplementing with exogenous VD, however, previous reports suggest 1000 IU VD3/day is a safe dosage (He et al., 2016).
• General VD guidelines for Northern hemisphere athletes: Aim to acquire 15 min of non-protected (i.e., no sunscreen) sun exposure per day (He et al., 2016).

### Heat Acclimation

The Summer Games are expected to be hot (>30°C) and humid (>70% relative humidity). Therefore, heat acclimation (HA) will be an integral component of the preparatory phase. Exercise immunology research suggests that heat does not pose a challenge to the immune system. Indeed, performing a one-off bout of exercise in hot conditions [28–38.7°C, 45–76% relative humidity (RH)] does not appear to exacerbate exercise induced immune perturbations, compared to temperate conditions (Mitchell et al., 2002; McFarlin and Mitchell, 2003; Niess et al., 2003; Laing S. et al., 2005; Laing S.J. et al., 2005). Similarly, HA has been shown to have negligible effects on immunity. For example, no change in white blood cell counts (Willmott et al., 2016) or inflammatory cytokines (Amorim et al., 2011; Barberio et al., 2015) has been demonstrated following HA. However, the current limitation to the HA studies discussed is that illness reports were not measured alongside immune markers. Therefore, it remains unclear how HA may impact upon athletes URTS risk. Consideration may want to be given to the acclimation status of athletes performing HA. During the preparatory phase, it is possible that acclimation status will differ between Northern and Southern hemisphere athletes; athletes residing in the Northern and Southern hemisphere will likely be seasonally acclimated and unacclimated, respectively. Future studies should assess the baseline acclimation status of athletes engaging in HA to understand whether it is associated with URTS risk.

#### Strategies to Maintain Athlete Health During HA

The health status of athletes should be considered when implementing HA. Athletes experiencing illness symptoms should not participate in HA as it may exacerbate illness (Casadio et al., 2017).

• Hygiene: Maintain good hygiene (e.g., remove wet clothing and have a warm shower immediately following HA sessions) (refer to Table 1 for further guidelines).
• Training load and recovery management: Heat stress adds to athletes overall training load. Carefully manage training load when training with additional heat (Walsh, 2018). Ensure adequate recovery between HA sessions, particularly if HA protocols involve prolonged exercise (≥90 min) as it can cause more severe immune perturbations than shorter duration exercise (<60 min) (Diment et al., 2015).
• Daily wellness monitoring and management: Monitor wellness to understand how individual athletes tolerate HA. A customized psychometric questionnaire (Hooper and Mackinnon, 1995) utilizing Likert scales can be used to assess indicators of wellness (e.g., sleep quality, stress, fatigue, mood, and muscle soreness) (Buchheit et al., 2013; Gallo et al., 2017). In addition to monitoring wellness during the HA period, the questionnaire should be administered during normal training weeks to establish baseline wellness data. High stress/anxiety levels and sleep deprivation have been linked to increased URTS incidence (Cohen et al., 1991, 2009). Over the HA period, apply strategies listed in Table 1 (i.e., minimize stress and anxiety and improve sleep). If athletes wellness scores are substantially reduced consider adjusting the HA protocol (e.g., reduce load) (Schwellnus et al., 2016).
• Carbohydrate (CHO) intake: Maintain day-to-day CHO availability over HA period, aim for >50% daily energy intake as CHO (Walsh, 2018).
• Hydration: Permissive dehydration is often used during HA sessions to accelerate the adaptation process (Garrett et al., 2014). Exercising in a dehydrated state does not appear to cause further exacerbation of immune perturbations, compared to euhydrated exercise (Svendsen et al., 2014; Killer et al., 2015). Therefore, permissive dehydration can be used during HA as it is unlikely to impair immunity. However, during recovery from HA, fluid replacement should be prioritized, as per current rehydration guidelines (Thomas et al., 2016).
• Probiotic supplementation: Begin supplementation at least 2-weeks before the HA block is due to commence and supplement throughout HA (refer to Table 1 for guidelines).

### Long Haul Travel

During the preparatory phase, many Southern and Northern hemisphere athletes will undertake international travel to competition, heat camps and the Summer Games itself. Travel has been identified as a prominent risk factor for URTS. Increased URTS incidence and severity has been demonstrated in team sport athletes traveling to international destinations that were >5 or 11 time zones, respectively (Haywood et al., 2014; Fowler et al., 2016). Similarly, in elite endurance athletes, air travel was found to significantly increase URTS susceptibility (Svendsen et al., 2016). The current limitation to the studies discussed is that immune markers were not measured alongside illness reports. Nevertheless, in the general population simulated long haul travel has been found to induce transient immune changes that may contribute to increased URTS susceptibility (Wilder-Smith et al., 2012). Contrastingly, a recent study in master-level athletes showed that long haul travel did not impair mucosal immune responses (Stevens et al., 2018). Further research on elite athletes is required to elucidate how travel impacts upon the immune system and subsequently URTS risk.

#### Strategies to Maintain Athlete Health During Long Haul Travel

• Travel vaccines: Consult a physician and update athlete and support staff vaccines.
• Hand hygiene: Apply alcohol-based hand gel after touching potentially contagious objects. For example, hand gel should be used after handling airport plastic security screening trays as a recent study identified that they have the highest frequency of respiratory viruses, compared to other airport surfaces (e.g., toilets, handrails) (Ikonen et al., 2018).
• Avoid ill people: If possible, seat athletes away from ill passengers. Increased risk for infection transmission has been associated with sitting within two rows of a contagious passenger for >8 h (Mangili and Gendreau, 2005). If it is not possible to change seats, athletes should wear a disposable face mask (Walsh et al., 2011a).
• Hydration: Encourage athletes to drink plenty of water to keep well hydrated and potentially prevent mucosal membranes from drying out.
• Optimize sleep hygiene: Pre-departure, improve sleep quantity and quality (refer to Table 1 for guidelines). After long haul travel, greatest sleep disruption has been reported in the first 48 h (Stevens et al., 2018). Optimize sleep hygiene (e.g., electronic device availability, cool room temperature, caffeine, ear plugs, eye masks, etc.) to improve sleep on the first 2 days after arrival (Stevens et al., 2018).
• Recovery: Avoid flying on the same day as competition or intensive training, delay travel until at least the subsequent day (Svendsen et al., 2016).
• Probiotic supplementation: Begin supplementation at least 2-weeks before scheduled travel (refer to Table 1 for guidelines).

## Competition Phase of the Summer Games

### Stressors Associated With the Summer Games

During the Summer Games, both Northern and Southern hemisphere athletes will be exposed to a range of stressors. Such stressors include, intensive competition, hot and humid environmental conditions, dehydration, psychological stress, and sleep deprivation (Keaney et al., 2018; Walsh, 2018). The effect of these stressors on immunity and illness risk has been summarized in recent reviews (Keaney et al., 2018; Walsh, 2018; Williams et al., 2018). Previous studies have tended to examine each stressor in isolation when in reality athletes will be simultaneously exposed to all stressors at the Summer Games. The synergism of these stressors could potentially have a compounding effect on immunodepression, resulting in higher incidence of illness than if each stressor were applied alone. Further research is needed to understand how multiple stressors affect immunity and illness risk.

At the Summer Games, medals are often won by the smallest of margins, so even a mild illness could negatively affect results. To keep athletes healthy and minimize the potential immunodepression evoked by Summer Games stressors, athletes should consider adhering to the five key illness prevention strategies listed in Table 1. These strategies have been selected on the assumption that Summer Games athletes will adhere to fundamental principles of nutrition and sport science (e.g., macro- and micro-nutrient intake, hydration, recovery protocols, training load management, etc.). Illness prevention strategies should not replace fundamentals, but work alongside them to keep athletes healthy. In addition to these strategies, other reviews exist which provide detailed recommendations on avoiding infection and maintaining immune health in athletes (Schwellnus et al., 2016; Walsh, 2018).

### Athletes at Increased Risk for Illness During the Summer Games

**FIGURE 1:**
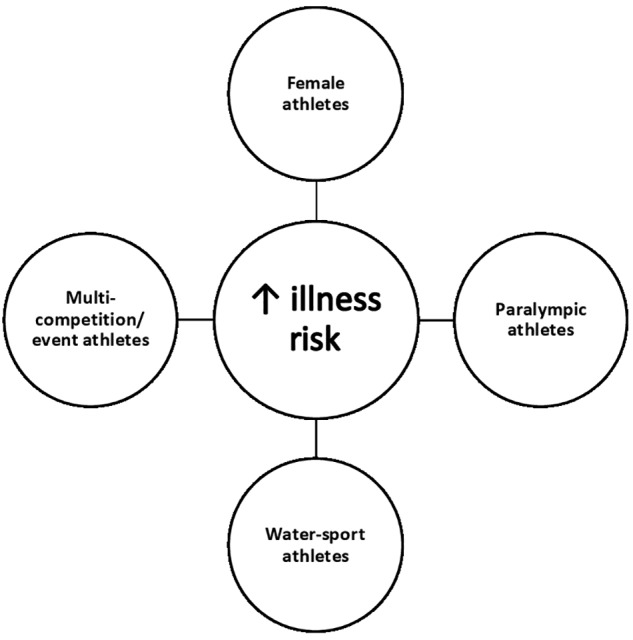
Athletes potentially at increased risk for URTS during the Summer Games.

With the aim of protecting the health of athletes, the International Olympic Committee (IOC) monitored illness incidence at the London (2012) and Rio (2016) Olympic and Paralympic Games (Derman et al., 2013, 2017; Engebretsen et al., 2013; Soligard et al., 2017). At previous Summer Games 5–14% of athletes experienced at least one illness, with the highest incidence of illness affecting the respiratory tract (Derman et al., 2013, 2017; Engebretsen et al., 2013; Soligard et al., 2017). IOC reports demonstrated that the illness rates varied considerably between gender and sports (Derman et al., 2013, 2017; Engebretsen et al., 2013; Soligard et al., 2017). As summarised in Figure 1, it appears that some athletes may be more susceptible to illness during the Summer Games, namely: (1) Female athletes; (2) Paralympic athletes; (3) Water-sport athletes; and (4) Multi-competition/event athletes (i.e., athletes who compete on >1 day) (Derman et al., 2013, 2017; Engebretsen et al., 2013; Soligard et al., 2017).

#### Female Athletes

Data obtained at the London and Rio Summer Games demonstrated significantly higher (40–60%) illness incidence in female compared to male athletes (Engebretsen et al., 2013; Derman et al., 2017; Soligard et al., 2017). In agreement with these findings, longitudinal studies have shown that female athletes tend to be at increased risk for URTS (Gleeson et al., 2011; He et al., 2014) and experience URTS episodes for a longer duration than male athletes (He et al., 2014). Sex differences in immune variables may explain the higher illness susceptibility observed in female athletes. Differences in immune responses between males and females have largely been attributed to sex hormones and their inherent immune modulatory functions (Klein and Flanagan, 2016). Furthermore, increased URTS susceptibility in female athletes may be associated with low energy availability (LEA). Higher rates of LEA have been demonstrated in female compared to male athletes (Logue et al., 2018), and LEA has been identified as a key risk factor for illness in Olympic-level female athletes (Drew et al., 2017).

##### Strategies to maintain female athlete health

• Diagnose and treat LEA: In the preparatory phase, identify female athletes with LEA using the validated questionnaire Low Energy Availability in Females Questionnaire (LEAF-Q) (Melin et al., 2014). Athletes with LEA should work closely with a nutritionist to ensure daily energy intake matches training and competition demands (Logue et al., 2018).
• Supplementation: At present, a number of supplements have been proposed to alter specific aspects of the immune system and reduce athletes URTS risk (Maughan et al., 2018). However, few supplements have convincing evidence supporting their use. Currently, probiotic (refer to Table 1 for guidelines), vitamin C (0.25–1.0 g/day) (Hemilä and Chalker, 2013) or quercetin (1 g/day) (Somerville et al., 2016) are the most promising supplements in this area, although further research is needed to determine how the combined use of these supplements influence URTS risk. Athletes need for these supplements should be assessed on an individual case-by-case basis, based on several factors (e.g., URTS history, sport, nutrient status, etc.). Supplements to be used at the Summer Games should be piloted (for acceptance/compliance/safety) in an off season/preparatory phase. Ensure selected supplements are batch tested and comply to WADA regulations.

#### Paralympic Athletes

Paralympic athletes appear to be more susceptible to illness than able-bodied athletes. Paralympic athletes suffered almost double the amount of URTS than able bodied athletes during previous London and Rio Summer Games (Paralympics: 12–14% vs. Able-bodied: 5–7%) (Derman et al., 2013, 2017; Engebretsen et al., 2013; Soligard et al., 2017). It is difficult to ascertain why Paralympic athletes are at a heightened risk for illness, as research on Paralympic sport is limited compared to investigations of able-bodied athletes (Van Rensburg et al., 2018). Illness risk will differ between Paralympic athletes based on their disability type. Paralympic athletes with spinal cord injuries have altered autonomic control and immunity, and impaired immune function has been cited as the main reason for increased illness susceptibly in this population (Leicht et al., 2013). In addition, the use of wheelchairs by Paralympic athletes likely increases infection transmission risk, as wheelchairs pick up and carry high numbers of bacteria. Indeed, at the Rio Paralympics, the highest illness incidence rate was reported in wheelchair fencing, while wheelchair basketball was only behind Paralympic swimming in terms of illness sustained (Derman et al., 2017).

##### Strategies to maintain paralympic athlete health

• Hygiene: Wheelchair athletes should regularly disinfect wheelchairs, wear gloves, ensure good hand hygiene, and avoid self-inoculation by touching eyes, nose, and mouth (Walsh, 2018).
• Supplementation (see section “Strategies to Maintain Female Athlete Health” for details).

#### Water-Sport Athletes

Athletes involved in Water-sports may be at an increased risk for illness during the Summer Games. At previous Summer Games, the IOC identified the top 5 sports with the highest illness incidence; water-sports accounted for 2 out of 5 (sailing and synchronized swimming) and 4 out of 5 (diving, open water marathon, canoe slalom, and synchronized swimming) sports at the London (Engebretsen et al., 2013) and Rio Olympics (Soligard et al., 2017), respectively. Similarly, at the Rio Paralympics, Para-Swimming was the sport with the second highest illness incidence (Derman et al., 2017). There are two likely factors underpinning increased illness susceptibility in this population: (1) chlorine exposure for pool-athletes; and (2) water quality issues for open-water athletes. Airway disorders, including asthma and rhinitis are prevalent in pool-athletes and are often attributed to chlorine and chlorine by-products causing airway changes (Škrgat et al., 2018). As such, asthma and allergy mediated non-infectious URTS may explain why pool-sport athletes appear to be at an increased risk for illness. Alternatively, for open-water sport athletes, particularly at the Rio Summer Games, reports suggest that water quality issues (i.e., contamination with bacteria and viruses) were the primary cause of higher illness incidence (Keith, 2017).

##### Strategies to maintain water-sport athlete health

• Diagnose and control asthma and allergies (see section “Strategies to Minimize Cold Air Mediated Non-infectious URTS” for details).
• Supplementation (see section “Strategies to Maintain Female Athlete Health” for details).

#### Multi-Competition/Event Athletes

Multi-competition/event athletes may be at an increased risk for illness. Indeed, data obtained at previous Olympic games demonstrated that of the top 5 sports with the highest illness incidence the majority were multi-competition/event sports [5/5 in London: Athletics, Beach VB, Football, Sailing, and Synchronized Swimming (Engebretsen et al., 2013)] [4/5 sports in Rio: Diving, Canoe Slalom, Equestrian and Synchronized Swimming (Soligard et al., 2017)]. It is unclear why these athletes are more susceptible to illness, nonetheless it may be explained by the psychological element of having to mentally prepare for multiple events. Recent research suggests that mental state influences immunity, for example, state-anxiety and perceived psychological stress before exercise has been shown to influence immune responses to a greater extent than exercise itself (Edwards et al., 2018). In addition, a significant association between mental health (i.e., perceived stress and depression) and illness incidence has been demonstrated in athletes preparing for the Rio Olympics (Drew et al., 2017). Further studies should explore mental state to better elucidate how it affects athletes’ immunity and URTS risk.

##### Strategies to maintain multi-competition/event athlete health

• Manage stress and anxiety (refer to Table 1 for guidelines).
• Mindfulness practices: Mindfulness interventions such as meditation, breathing awareness, walking and yoga have the potential to alleviate psychological stress and anxiety. Recent studies have reported significant improvements to athletes’ mental state with 4–6 weeks of mindfulness training (Ajilchi et al., 2019; Chen et al., 2019). Furthermore, in wheelchair basketball players, 8 weeks of mindful mediation utilizing a smart phone app attenuated the rise in cortisol associated with a competition period (MacDonald and Minahan, 2018). However, immune responses did not appear to be influenced (MacDonald and Minahan, 2018). It is currently unclear if mindful training influences URTS risk in athletes, nevertheless in the general population a reduction in URTS incidence has been demonstrated following 8 weeks of mindful meditation (Barrett et al., 2012). Mindfulness training appears to be a promising strategy for athletes, although further investigation is warranted. Athletes planning to use mindfulness interventions at the Summer Games should pilot and optimize practices in an off season/preparatory period.
• Supplementation (see section “Strategies to Maintain Female Athlete Health” for details).

## Conclusion

It is apparent that athletes will be exposed to various stressors during both the preparatory and competition phases of the Summer Games. Athletes residing in the southern hemisphere appear to be at increased risk for illness during the preparatory phase, while female, Paralympic, water-sport and multi-competition/event athletes may be more susceptible to illness during the competition phase of the Summer Games. To maintain athlete health, illness prevention strategies should be targeted to stressors and at-risk athletes. Keeping athletes healthy will contribute to optimal Olympic and Paralympic athletic performance. While the considerations and strategies outlined in this mini review are targeted for the Summer Games, many could be used for other major competitions and as such should be considered for future sporting success.

## Author Contributions

All authors were involved in the conception of the manuscript. LK drafted the manuscript. FM, AK, and DD critically revised the manuscript and approved the final version to be published.

## Conflict of Interest Statement

The authors declare that the research was conducted in the absence of any commercial or financial relationships that could be construed as a potential conflict of interest.
